# Genome Characterization of a Pathogenic Porcine Rotavirus B Strain Identified in Buryat Republic, Russia in 2015

**DOI:** 10.3390/pathogens7020046

**Published:** 2018-04-20

**Authors:** Konstantin P. Alekseev, Aleksey A. Penin, Alexey N. Mukhin, Kizkhalum M. Khametova, Tatyana V. Grebennikova, Anton G. Yuzhakov, Anna S. Moskvina, Maria I. Musienko, Sergey A. Raev, Alexandr M. Mishin, Alexandr P. Kotelnikov, Oleg A. Verkhovsky, Taras I. Aliper, Eugeny A. Nepoklonov, Diana M. Herrera-Ibata, Frances K. Shepherd, Douglas G. Marthaler

**Affiliations:** 1 N. F. Gamaleya National Research Center for Epidemiology and Microbiology, Gamaleya Str. 18, Moscow 123098, Russia; amuhin@yahoo.com (A.N.M.); t_grebennikova@mail.ru (T.V.G.); anton_oskol@mail.ru (A.G.Y.); aliper@narvac.com (T.I.A.); 2 Federal State Budget Scientific Institution “Federal Scientific Centre VIEV”, Moscow 109428, Russia; raevsergey@mail.ru; 3 A. N. Belozersky Institute of Physico-Chemical Biology, Lomonosov Moscow State University, Moscow 119991, Russia; alekseypenin@gmail.com; 4 Institute for Information Transmission Problems of the Russian Academy of Sciences, Moscow 127051, Russia; 5 Laboratory of Extreme Biology, Institute of Fundamental Biology and Medicine, Kazan Federal University, Kazan 420021, Russia; 6 Department of Genetics, Faculty of Biology, Lomonosov Moscow State University, Moscow 119991, Russia; 7 Independent Non-Profit Organization “Diagnostic and Prevention Research Institute for Human and Animal Diseases”, Moscow 123098, Russia; kizkhalum@yandex.ru (K.M.K.); annamoskvina17@gmail.com (A.S.M.); m_vovk@list.ru (M.I.M.); doktor-mishin@mail.ru (A.M.M.); apkotelnikov@yandex.ru (A.P.K.); info@dpri.ru (O.A.V.); 8 The Ministry of Agriculture of the Russian Federation, Orlikov Pereulok 1/11, Moscow 107139, Russia; pr.nepoklonova@mcx.ru; 9 Veterinary Diagnostic Laboratory, College of Veterinary Medicine, Kansas State University, 1800 Denison Ave, Manhattan, KS 66502, USA; dianaherrera@vet.k-state.edu; 10 Department of Veterinary and Biomedical Sciences, College of Veterinary Medicine, University of Minnesota, St. Paul, MN 55108, USA; sheph085@umn.edu

**Keywords:** porcine group B rotavirus, RVB, gastrointestinal disease, porcine enteric disease, phylogenetic analysis

## Abstract

An outbreak of enteric disease of unknown etiology with 60% morbidity and 8% mortality in weaning piglets occurred in November 2015 on a farm in Buryat Republic, Russia. Metagenomic sequencing revealed the presence of rotavirus B in feces from diseased piglets while no other pathogens were identified. Clinical disease was reproduced in experimentally infected piglets, yielding the 11 RVB gene segments for strain Buryat15, with an RVB genotype constellation of G12-P[4]-I13-R4-C4-M4-A8-N10-T4-E4-H7. This genotype constellation has also been identified in the United States. While the Buryat15 VP7 protein lacked unique amino acid differences in the predicted neutralizing epitopes compared to the previously published swine RVB G12 strains, this report of RVB in Russian swine increases our epidemiological knowledge on the global prevalence and genetic diversity of RVB.

## 1. Introduction

Rotaviruses (RVs) were first isolated in 1973 from children in Australia [1,2]. After the identification in swine two years later [3], RVs were recognized as the major etiological agents of acute viral gastroenteritis in humans and domesticated livestock worldwide [4,5,6]. Belonging to the *Reoviridae* family, the RV genome is composed of 11 double stranded RNA segments [7]. Eight RV species (RVA-RVH) and two tentative species (RVI and RVJ) have been identified by sequence-based classification of inner capsid protein 6 (VP6) [8,9,10]. RVA, RVB, RVC and RVH have been detected in both humans and animals while RVD-RVG, RVI, and RVJ have only been found in animals. Five out of ten RV species have been described in pigs (RVA, RVB, RVC, RVE, and RVH) [11,12,13].

Of the RV species, RVA is most common and well characterized both in animals and humans due to its high prevalence and pathogenicity. Porcine RVA was isolated in 1975 [3] followed by identification of swine RVC [14] and RVB [15,16]. A recent two-year study found RVB in 31.8% of diarrheic samples from North American swine, indicating higher detection of RVB than previously observed [16,17]. Similar detection rates of porcine RVB (25.9%) have been identified in Japan [18]. Although identified at lower rates than in North America and Japan, swine RVB has also been detected in Europe, South Africa, India, and Brazil [19,20,21,22]. 

Despite unexpectedly high detection rates of RVB in swine, RVB pathogenesis has only been established in gnotobiotic and caesarian-derived colostrum-deprived piglets [16,23]. The inability to cultivate RVB and limited whole genome sequence data has hampered an understanding of transmission and evolution within pigs. In order to fill these knowledge gaps, this study used metagenomic sequencing to identify porcine RVB from an enteric outbreak in a farm from southern Siberia, determined its disease-causing ability using experimental inoculation experiments, and studied its phylogenetic relationship with previously characterized swine RVB strains.

## 2. Results

During late autumn 2015, an outbreak of enteric disease occurred in three-day old suckling piglets on a farm located in Buryat Republic, Russia. Approximately 60% of litters had watery diarrhea (lasting 3–5 days), and the mortality rate was approximately 8%. The surviving piglets had reduced weight gain and a delay of being sent to market. Fecal and intestinal samples from infected piglets were submitted to the Diagnostic and Prevention Research Institute for Human and Animal Diseases to identify the cause of the disease. The samples tested negative for TGEV (Transmissible Gastroenteritis Virus), RVA, ASFV (African Swine Fever Virus), PCV-2 (Porcine Circovirus Type 2), CSFV (Classical Swine Fever Virus), and PRCV (Porcine Respiratory Coronavirus) using ELISA and PCR commercial kits from Vetbiochim (Moscow, Russia). Fecal samples were passaged on Vero, ST, and PK-15. Cell culture was halted after six blind passages since the cytopathic effect (CPE) was not observed. The fecal and intestinal samples were negative for bacterial pathogens on blood agar plates. Since a pathogen was not identified as the causative agent of disease, the purified RNA from the intestinal samples were submitted for Next Generation Sequencing (NGS). De novo assembly of the reads generated two contigs, which upon BLAST (NCBI) analysis yielded 83% and 86% nucleotide identities to the VP3 and VP4 genes of porcine RVB strain LS00011_Ohio, respectively. No other pathogens were detected in the NGS data.

A piglet was infected with the filtered fecal material and commingled with a mock-inoculated piglet to investigate the etiology and transmission associated with porcine RVB strain Buryat15. The fecal infected piglet developed diarrhea within 12 h while the mock-inoculated piglet developed diarrhea 24 h post-inoculation (PI) due to being commingled with the infected piglet. The small and large intestinal homogenates from the two pigs tested negative by the previously described commercial ELISA and PCR kits from Vetbiochim. Passage of the small and large intestinal homogenates in the Vero, ST, and PK-15 cells lacked CPE after six passages. Testing of the intestinal homogenates by NGS identified the addition of nine gene segments of RVB strain Buryat15. NGS did not identify any other pathogens in the intestinal homogenates.

**Figure 1:**
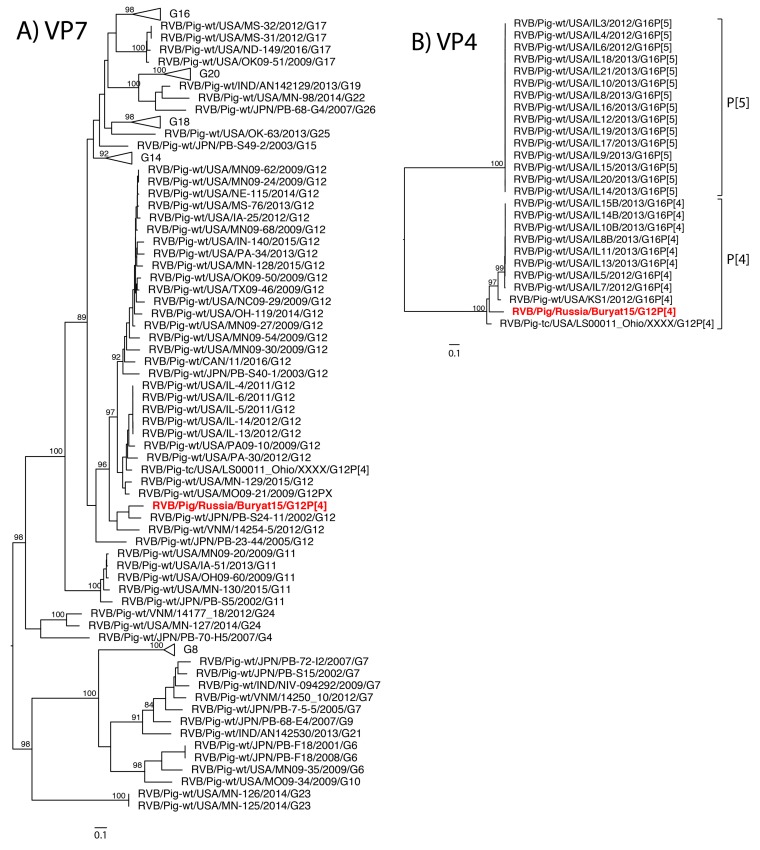

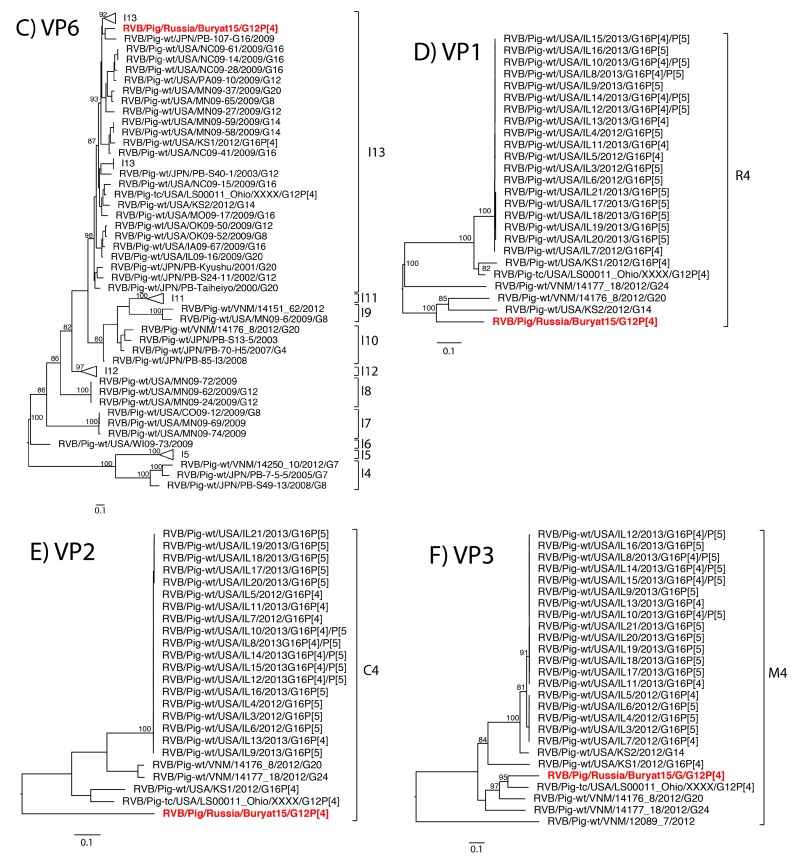

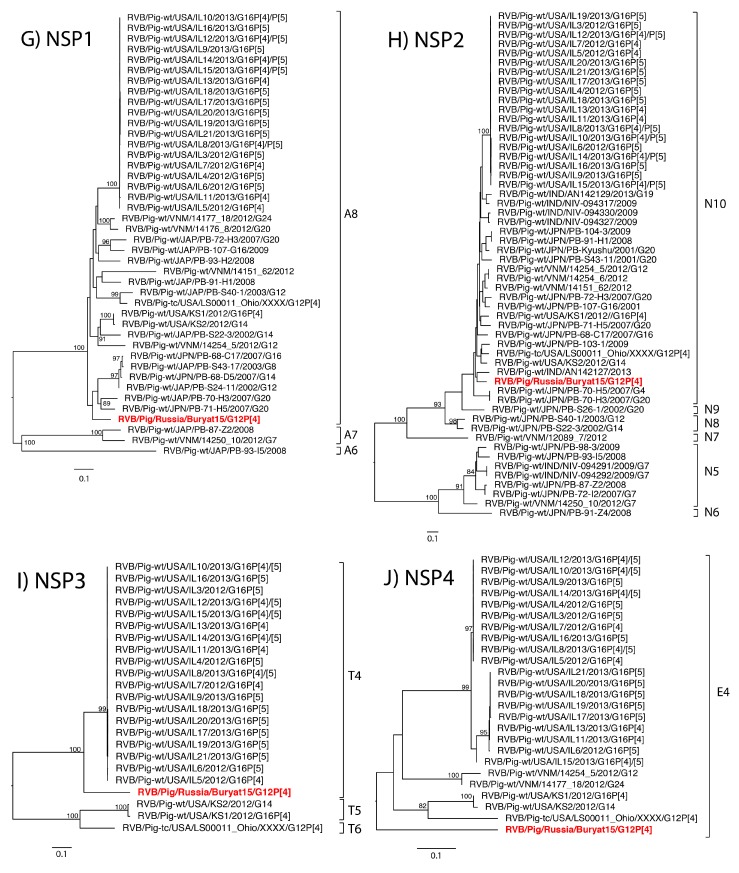

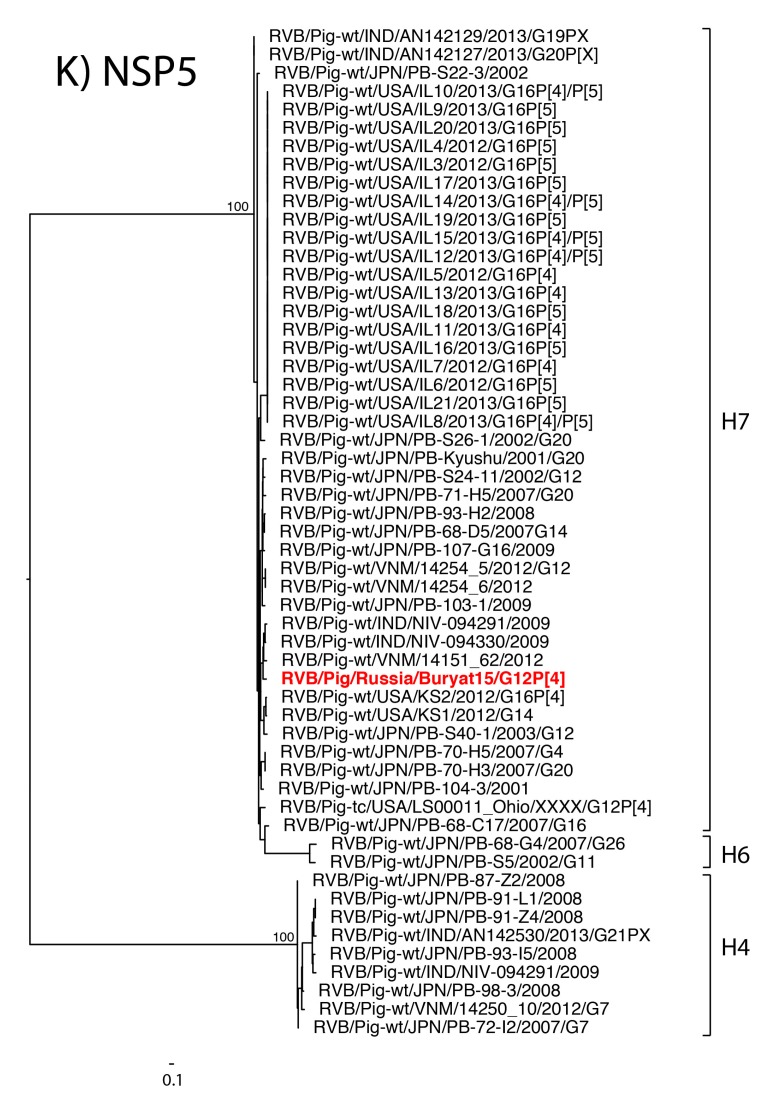
Phylogenetic trees for the 11 gene segments of swine RVB (**A**–**K**) with bootstrap values represented at the nodes (500 replicates). Bootstrap values below 80% are not shown. Selected genotype clades were clasped and represented by triangles. Russian strain Buryat15 is represented in bolded red. Scale bars represent 10 nucleotide changes per nucleotide site. Genotypes are labeled with brackets for all strains except VP7, where G genotypes are listed in the strain name.

The eleven gene segments of Buryat15 had the highest nucleotide identities with genes of RVB available through GenBank and were assigned a genotype constellation of G12-P[4]-I13-R4-C4-M4-A8-N10-T4-E4-H7 based on the whole RVB genome nucleotide cutoff values proposed by Shepherd et al. (manuscript in review). Thus, phylogenetic analysis focused on comparison with strains of porcine origin. Phylogenetic analysis of strain Buryat15 revealed a porcine ancestry of mixed geographic origins (Figure 1A–K).

The VP7 gene shared close common ancestors with Japanese strains while the NSP2 gene was most closely related to a porcine strain from India. The VP6 gene branched with Japanese strain PB-107-G16, but both fell within a larger clade of United States strains. The NSP5 gene was most closely related to the cogent gene of a porcine strain from Vietnam. The VP1 gene shared a clade with swine RVB strains from Vietnam and United States. The NSP3 gene shared a large clade with swine RVB strains from the United States. The tissue culture adapted strain USA/LS00011_Ohio was closely related to the Buryat15 VP3 gene segment. The VP4, VP2, and NSP4 genes from Buryat15 lacked close neighbors in the phylogenetic trees. 

To explore the antigenic diversity of Buryat15, the VP7 amino acid identities were compared to previously characterized swine RVB strains of the G12 genotype at predicted antigenic sites [24] (Table 1). Buryat15 has an asparagine at the hypervariable residue 65, which is only shared by RVB strains isolated in Illinois, USA. Strains Buryat15 and Japanese PB-S24-11 have a glutamic acid at residue 91 while all the RVB strains have an alanine residue.

## 3. Discussion

Limited information is available for non-RVA species in human and domesticated livestock from Russia. A single manuscript described RVC in humans from the Novosibirsk and Omsk regions of Russia [25] while RVB in Russia has so far not been reported. Until recently, RVB was not considered an important pathogen in pigs, although early research demonstrated pathogenesis of RVB in gnotobiotic piglets [16]. While RVC infections are common in neonatal piglets, outbreaks of RVB in neonatal piglets are not typically reported as RVB infections are predominantly identified in older pigs [11]. Our results indicate that RVB is capable of causing enteric disease in conventionally raised, neonatal piglets and highlight the ability of RVB to cause and replicate clinical disease in piglets. These results further improve our understanding of the complexity associated with RVB as a swine pathogen.

The clinical disease reproduced by in conventionally raised piglets induced severe watery diarrhea 12 h PI. While the samples were negative for other bacterial and viral pathogens by traditional detection methods and NGS, it is still possible that RVB infection may cause disease in concert with other RV species or bacteria. However, several factors suggest RVB was the causative agent of enteric disease in the piglets. First, no other pathogens were identified in the NGS data, suggesting that RVB was the main disease-causing pathogen in the sample. Moreover, the purified fecal sample used to infect the piglet did not contain bacteria, and RVB RNA was detected in the mock-inoculated, commingled piglet, suggesting transmission of RVB. However, IHC staining or in situ hybridization is necessary to confirm this hypothesis and were not available at the time of the study.

RVB strains from different host species are genetically different from one another [26,27,28], which was consistent with our analysis since Buryat15 had the highest nucleotide identity with swine RVB strains. The long branches with Buryat15 in the VP2, VP4, and NSP4 phylogenetic trees indicate a lack of information on the genetic diversity of porcine RVB strains. The Buryat15 gene segments clustered closely with porcine RVB strains from different countries including Vietnam, India, Japan, and the United States. Reassortment is a common event within RV species [27,29,30,31], and swine RVB strains from India and the United States share recent common ancestors with Japanese porcine RVB strains [20,31]. A similar genetic relationship between Japanese and North American swine RVC has been demonstrated as well [32]. 

The genotype constellation of Buryat15 has not been identified in swine before but is closely related to constellations previously identified in swine from the United States (Shepherd et al., manuscript in review). Buryat15 and tissue culture strain LS00011_Ohio share the same genotypes for all genes except for NSP3 (T4 versus T6, respectively) while Buryat15 and United States strains IL11, IL13, IL5, and IL7 share genotypes for all genes besides VP7. The Russian strain was also related to United States swine based on its similarity at the predicted antigenic site to swine RVB strains from the USA [24]. However, the number of RVB gene segments available for comparison is limited, especially for several of the NSP gene segments, and a finer resolution of RVB evolution and antigenic diversity may be obtained with sequencing additional RVB strains.

Although the NGS data strongly suggest that RVB was responsible for the diarrhea outbreak in the Buryat Republic, immunofluorescent staining for the RVB antigens and in situ hybridization of the nucleic acid in fixed enterocytes from clinical and experimental animals would confirm infection and viral replication. Nevertheless, this study demonstrates the ability of an RVB strain to cause disease within conventionally raised piglets and illustrates the potential of reassortment within the evolutionary history of RVB in swine. Future epidemiological studies should be performed to continue characterizing the prevalence and diversity of Russian and global RVB strains in swine.

## 4. Materials and Methods

In November 2015, severe watery diarrhea was reported in newborn piglets (3–5 days of age). Samples were submitted to Diagnostic and Prevention Research Institute for Human and Animal Diseases for diagnostic testing. ELISA and PCR diagnostic kits were used to detect the following: TGEV and RVA, ELISA; ASFV, ELISA and PCR; PCV2, PCR; CSFV, PCR; and TGEV/PRCV, PCR; were used according to manufacturer recommendation (Vetbiochim, Moscow, Russia). RNA extraction was performed with GeneJET Viral DNA/RNA Purification Kit (Thermo Scientific, Waltham, MA, USA). Once RVB was identified by NGS, subsequent samples were tested using RVB primers by PCR (VP4-I-823-842-F: CGTATCCAAAGCCAACGGGA and VP4-I-1008-1028-R: TGGGCCCTTATTTTCCAGTGT), which were designed using the primer-BLAST online software and Buryat15 VP4 sequence KU744407. Synthesis of cDNA was performed according to a random primer protocol using RevertAid H Minus First Strand cDNA Synthesis Kit (Thermo Scientific). PCR was carried out using True-Start DNA polymerase with 10 mM dNTPs mix (Thermo Scientific) according to the manufacturer’s protocols.

Fecal samples from diarrheic piglets were diluted 1:10 in minimal essential medium (MEM) containing 1% actinomycin and 1% non-essential amino acids (Gibco, Grand Island, NY, USA) and clarified by centrifugation. Supernatants were filtered through 0.8 µm, 0.45 µm and 0.2 µm syringe filters sequentially, serial diluted, and added to five-day old monolayers of Vero, Swine testicular cells (ST) and porcine kidney 15 (PK-15). Six blind passages were performed with the cell lines and supernatants were saved at −70 °C.

To establish pathogenicity of the unknown virus, a single ten-day-old conventionally raised piglet was infected with 0.2 µm filtered, 1:10 diluted fecal sample while a single piglet was mock-infected with MEM. The two piglets were comingled to demonstrate transmission of pathological agent between piglets under experimental conditions. The sample was tested for the absence of bacterial contamination by seeding the filtered dilutions on blood agar plates. Piglets were monitored every 30 min for clinical signs of diarrhea. The piglets were sacrificed after the onset of diarrhea, and intestinal samples were collected. 

For NGS, previously extracted RNA underwent cDNA synthesis according to random primer protocol was performed on RevertAid H Minus First Strand cDNA Synthesis Kit (Thermo scientific). PCR was carried out using True-Start DNA polymerase with 10 mM dNTPs mix and 10 pmol specific primers per reaction (Thermo Scientific), according to manufacturer’s protocols. TruSeq Stranded Total RNA Library Prep Kit was used with 1 μg total RNA for the construction of libraries according to the manufacturer’s protocol. For rRNA-depleted library, rRNA was removed from 2.5 μg total RNA using Ribo-Zero rRNA Removal Kit (mixture 1:1 Human/Mouse/Rat probe and Bacteria probe), according to the manufacturer’s protocol (with probe concentration for epidemiology kit protocol). All cDNA libraries were sequenced using an Illumina HiSeq2000 (Illumina, San Diego, CA, USA), producing 101 × 7 × 101 bp paired-end reads with multiplexing. Reads were trimmed using default parameters with CLC Genomics Workbench 8.5.1 (Qiagen Bioinformatics, Redwood City, CA, USA). Trimmed reads were de novo assembled using a word size of 64, bubble size of 100, and minimum contig length of 300. The contigs were subject to the BLASTN search. RVB sequences were deposited into GenBank with the accession numbers KU744406 (VP3), KU744407 (VP4), KX869730-KX869737 (VP1, VP6, VP7, NSP1-NSP5), and MH093644 (VP2).

The newly generated RVB sequences were aligned using MUSCLE in Geneious (version 9.6.1, Newark, NJ, USA) [33] with porcine RVB sequences that had at least 80% of the open reading frame available in GenBank (Supplementary Tables S1–S11). Maximum likelihood phylogenetic trees were made with the RAxML method using a Generalized Time Reversible gamma model of nucleotide substitution and 500 bootstrap replicates. The swine RVB G12 strains were translated and aligned with Buryat15 to compare the 21 predicted antigenic sites of RVB VP7 [24].

## Figures and Tables

**Table 1:** Comparison of the predicted antigenic sites on VP7 [24] for swine RVB G12 strains. Dots represent the same residues compared to the consensus, determined by the majority amino acid residue of the alignment.

Predicted Epitope Location	33	34	36	37	39	40	65	66	67	89	90	91	92	130	158	159	160	161	179	180	181
Consensus	D	D	N	D	K	Q	X	N	Y	K	Y	A	Y	D	P	D	R	R	S	N	N
Russia/Buryat15/2015	**.**	**.**	**.**	**.**	**.**	**.**	N	**.**	**.**	**.**	**.**	E	**.**	**.**	**.**	**.**	**.**	**.**	**.**	**.**	**.**
JPN/PB-S24-11/2002	**.**	**.**	**.**	**.**	E	**.**	D	**.**	**.**	**.**	**.**	E	**.**	**.**	**.**	**.**	**.**	**.**	**.**	**.**	**.**
JPN/PB-S40-1/2003	**.**	**.**	T	E	**.**	**.**	V	S	**.**	**.**	**.**	**.**	**.**	**.**	**.**	**.**	**.**	**.**	**.**	**.**	**.**
CAN/11/2016	**.**	**.**	T	**.**	**.**	**.**	D	Q	**.**	**.**	**.**	**.**	**.**	**.**	**.**	N	**.**	**.**	**.**	S	**.**
USA/PA-30/2012	**.**	**.**	T	**.**	**.**	K	D	**.**	**.**	**.**	**.**	**.**	**.**	**.**	**.**	**.**	**.**	**.**	**.**	**.**	**.**
USA/MN-129/2015	**.**	**.**	T	**.**	**.**	K	D	**.**	**.**	**.**	**.**	**.**	**.**	**.**	**.**	**.**	**.**	**.**	**.**	**.**	**.**
USA/MN-128/2015	**.**	**.**	**.**	**.**	**.**	**.**	E	**.**	**.**	**.**	**.**	**.**	**.**	**.**	**.**	N	**.**	**.**	**.**	**.**	**.**
USA/IL-13/2012	**.**	**.**	T	**.**	**.**	K	N	D	**.**	**.**	**.**	**.**	**.**	**.**	**.**	**.**	**.**	**.**	**.**	**.**	**.**
USA/IL-5/2011	**.**	**.**	T	**.**	**.**	K	N	D	**.**	**.**	**.**	**.**	**.**	**.**	**.**	**.**	**.**	**.**	**.**	**.**	**.**
USA/IL-6/2011	**.**	**.**	T	**.**	**.**	K	N	D	**.**	**.**	**.**	**.**	**.**	**.**	**.**	**.**	**.**	**.**	**.**	**.**	**.**
USA/IL-4/2011	**.**	**.**	T	**.**	**.**	K	N	D	**.**	**.**	**.**	**.**	**.**	**.**	**.**	**.**	**.**	**.**	**.**	**.**	**.**
USA/IL-14/2012	**.**	**.**	T	**.**	**.**	K	N	D	**.**	**.**	**.**	**.**	**.**	**.**	**.**	**.**	**.**	**.**	**.**	**.**	**.**
USA/IN-140/2015	**.**	**.**	**.**	**.**	**.**	**.**	G	**.**	**.**	**.**	**.**	**.**	**.**	**.**	**.**	**.**	**.**	**.**	**.**	**.**	**.**
USA/OH-119/2014	**.**	**.**	**.**	**.**	**.**	**.**	Q	**.**	**.**	**.**	**.**	**.**	**.**	**.**	**.**	**.**	**.**	**.**	**.**	**.**	**.**
USA/PA-34/2013	**.**	**.**	**.**	**.**	**.**	**.**	E	**.**	**.**	**.**	**.**	**.**	**.**	**.**	**.**	N	**.**	**.**	**.**	**.**	**.**
USA/IA-25/2012	**.**	**.**	**.**	**.**	**.**	**.**	D	**.**	**.**	**.**	**.**	**.**	**.**	**.**	**.**	N	**.**	**.**	**.**	**.**	**.**
USA/NE-115/2014	**.**	**.**	**.**	**.**	**.**	**.**	E	**.**	**.**	**.**	**.**	**.**	**.**	**.**	**.**	N	**.**	**.**	**.**	**.**	**.**
USA/MS-76/2013	**.**	**.**	**.**	**.**	**.**	**.**	E	**.**	**.**	**.**	**.**	**.**	**.**	**.**	**.**	N	**.**	**.**	**.**	**.**	**.**
USA/PA09-10/2009	**.**	**.**	T	**.**	**.**	K	D	**.**	**.**	**.**	**.**	**.**	**.**	**.**	**.**	**.**	**.**	**.**	**.**	**.**	**.**
USA/MO09-21/2009	**.**	**.**	T	**.**	**.**	K	D	**.**	**.**	**.**	**.**	**.**	**.**	**.**	**.**	**.**	**.**	**.**	**.**	**.**	**.**
USA/OK09-50/2009	**.**	**.**	**.**	**.**	**.**	**.**	E	**.**	**.**	**.**	**.**	**.**	**.**	**.**	**.**	N	**.**	**.**	**.**	**.**	**.**
USA/MN09-54/2009	**.**	**.**	**.**	**.**	**.**	**.**	Q	**.**	**.**	**.**	**.**	**.**	**.**	**.**	**.**	**.**	**.**	**.**	**.**	S	**.**
USA/MN09-30/2009	**.**	**.**	**.**	**.**	**.**	**.**	I	**.**	**.**	**.**	**.**	**.**	**.**	**.**	**.**	**.**	**.**	**.**	**.**	**.**	**.**
USA/MN09-27/2009	**.**	**.**	**.**	**.**	**.**	**.**	Q	**.**	**.**	**.**	**.**	**.**	**.**	**.**	**.**	**.**	**.**	**.**	**.**	**.**	**.**
USA/MN09-24/2009	**.**	**.**	**.**	**.**	**.**	**.**	E	**.**	**.**	**.**	**.**	**.**	**.**	**.**	**.**	N	**.**	**.**	**.**	**.**	**.**
USA/MN09-68/2009	**.**	**.**	**.**	**.**	**.**	**.**	E	**.**	**.**	**.**	**.**	**.**	**.**	**.**	**.**	N	**.**	**.**	**.**	**.**	**.**
USA/LS00011_Ohio/XXXX	**.**	**.**	T	**.**	**.**	K	S	D	**.**	**.**	**.**	**.**	**.**	**.**	**.**	**.**	**.**	**.**	**.**	**.**	**.**

## Supplementary Materials

The following are available online at http://www.mdpi.com/2076-0817/7/2/46/s1, Tables S1–S11: Genbank accession numbers of swine RVB strains used in phylogenetic analysis.
