# Supplementary material for: Genome Characterization of a Pathogenic Porcine Rotavirus B Strain Identified in Buryat Republic, Russia in 2015
Source: Pathogens. 2018 Apr 20;7(2):46. doi: 10.3390/pathogens7020046 (PMC6027140; doi:10.3390/pathogens7020046)
Supplement: Supplementary file 1 [file pathogens-07-00046-s001.pdf]

Table S1. Genbank accession numbers of swine NSP1 used in phylogenetic analysis with Buryat15.

AB64632.1	AB646357.1	KX362395.1	MF966606.1	MF966610.1
KX362417.1	KX362428.1	MF966597.1	MF966605.1	MF966609.1
KR052709.1	KX362439.1	MF966598.1	MF966604.1	AB646361.1
AB646352.1	MF966616.1	MF966602.1	MF966608.1	AB646360.1
AB646356.1	MF966617.1	MF966601.1	MF966615.1	KX362384.1
AB646353.1	AB646350.1	MF966600.1	MF966614.1	AB646363.1
AB646354.1	AB646364.1	MF966599.1	MF966613.1	
AB646351.1	AB646358.1	MF966603.1	MF966612.1	
AB646355.1	KX362406.1	MF966607.1	MF966611.1	

Table S2. Genbank accession numbers of swine NSP2 used in phylogenetic analysis with Buryat15.

KR052710.1	AB673218.1	AB673231.1	MG272006.1	MG271998.1
KJ613656.1	AB673219.1	AB673215.1	MG271993.1	MG272005.1
KJ613657.1	AB673220.1	AB673216.1	MG271994.1	MG271987.1
KJ613651.1	AB673221.1	AB673217.1	MG271995.1	MG271986.1
KJ613651.1	AB673222.1	AB673229.1	MG271996.1	KX362374.1
KJ613653.1	AB673226.1	MG272003.1	MG271997.1	KX362385.1
KJ613654.1	AB673227.1	MG271999.1	MG271988.1	KX362418.1
KJ613655.1	AB673223.1	MG271992.1	MG271989.1	KX362429.1
AB673224.1	AB673230.1	MG272000.1	MG271990.1	KX362440.1
AB673225.1	AB673233.1	MG272004.1	MG272002.1	
AB673232.1	AB673228.1	MG272001.1	MG271991.1	

Table S3. Genbank accession numbers of swine NSP3 used in phylogenetic analysis with Buryat15.

KR052711.1	MG272019.1	MG272024.1	MG272010.1	MG272027.1
MG272015.1	MG272020.1	MG272025.1	MG272011.1	MG272028.1
MG272016.1	MG272021.1	MG272026.1	MG272012.1	
MG272017.1	MG272022.1	MG272008.1	MG272013.1	
MG272018.1	MG272023.1	MG272009.1	MG272014.1	

Table S4. Genbank accession numbers of swine NSP4 used in phylogenetic analysis with Buryat15.

KR052712.1	MG272041.1	MG272046.1	MG272032.1	MG272049.1
MG272037.1	MG272042.1	MG272047.1	MG272033.1	MG272050.1
MG272038.1	MG272043.1	MG272048.1	MG272034.1	KX362409.1
MG272039.1	MG272044.1	MG272030.1	MG272035.1	KX362431.1
MG272040.1	MG272045.1	MG272031.1	MG272036.1	

Table S5. Genbank accession numbers of swine NSP5 used in phylogenetic analysis with Buryat15.

KR052713.1	AB713982.1	AB713992.1	MG272063.1	MG272055.1
KJ613663.1	AB713983.1	AB713975.1	MG272064.1	MG272056.1
KJ613664.1	AB713984.1	AB713977.1	MG272065.1	MG272057.1
KJ613665.1	AB713985.1	AB713978.1	MG272066.1	MG272058.1
KJ613660.1	AB713986.1	AB713979.1	MG272067.1	MG272071.1
KJ613661.1	AB713990.1	AB713980.1	MG272068.1	MG272072.1
KJ613662.1	AB713991.1	AB713976.1	MG272069.1	KX362388.1
AB713988.1	AB713993.1	MG272059.1	MG272070.1	KX362421.1
AB713989.1	AB713994.1	MG272060.1	MG272052.1	KX362432.1
AB713974.1	AB713987.1	MG272061.1	MG272053.1	KX362443.1
AB713981.1	AB713973.1	MG272062.1	MG272054.1	

Table S6. Genbank accession numbers of swine VP1 used in phylogenetic analysis with Buryat15.

KR052714.1	MG272084.1	MG272089.1	MG272075.1	MG272092.1
MG272080.1	MG272085.1	MG272090.1	MG272076.1	MG272093.1
MG272081.1	MG272086.1	MG272091.1	MG272077.1	KX362389.1
MG272082.1	MG272087.1	MG272073.1	MG272078.1	KX362400.1
MG272083.1	MG272088.1	MG272074.1	MG272079.1	

Table S7. Genbank accession numbers of swine VP2 used in phylogenetic analysis with Buryat15.

KR052715.1	MG272106.1	MG272111.1	MG272097.1	MG272114.1
MG272102.1	MG272107.1	MG272112.1	MG272098.1	KX362390.1
MG272103.1	MG272108.1	MG272113.1	MG272099.1	KX362401.1
MG272104.1	MG272109.1	MG272095.1	MG272100.1	
MG272105.1	MG272110.1	MG272096.1	MG272101.1	

Table S8. Genbank accession numbers of swine VP3 used in phylogenetic analysis with Buryat15.

KR052716.1	MG272126.1	MG272131.1	MG272117.1	MG272134.1
MG272122.1	MG272127.1	MG272132.1	MG272118.1	MG272135.1
MG272123.1	MG272128.1	MG272133.1	MG272119.1	KX362369.1
MG272124.1	MG272129.1	MG272115.1	MG272120.1	KX362391.1
MG272125.1	MG272130.1	MG272116.1	MG272121.1	KX362402.1

Table S9. Genbank accession numbers of swine VP4 used in phylogenetic analysis with Buryat15.

KR052717.1	MG272150.1	MG272155.1	MG272160.1	MG272141.1
MG272145.1	MG272151.1	MG272156.1	MG272137.1	MG272142.1
MG272146.1	MG272152.1	MG272157.1	MG272138.1	MG272143.1
MG272147.1	MG272153.1	MG272158.1	MG272139.1	MG272144.1
MG272148.1	MG272154.1	MG272159.1	MG272140.1	MG272161.1

Table S10. Genbank accession numbers of swine VP6 used in phylogenetic analysis with Buryat15.

KR052718.1	KF882576.1	MG272168.1	KF882574.1	KF882564.1
KF882592.1	KF882572.1	MG272169.1	KF882533.1	KF882565.1
KF882585.1	KF882575.1	KF882559.1	KF882516.1	KF882567.1
KF882591.1	KF882575.1	KF882560.1	KF882527.1	KF882569.1
KF882590.1	KF882579.1	MG272182.1	KF882571.1	KF882568.1
KF882580.1	MG272170.1	MG272183.1	KF882548.1	KF882570.1
KF882581.1	MG272171.1	KF882529.1	KF882554.1	KF882536.1
KF882582.1	MG272172.1	KF882534.1	KF882554.1	KF882531.1
KF882583.1	MG272173.1	KF882540.1	KF882573.1	KF882537.1
KF882584.1	MG272174.1	KF882541.1	KF882562.1	KF882546.1
KF882586.1	MG272175.1	KF882552.1	KF882524.1	KF882553.1
KF882587.1	MG272176.1	KF882561.1	KF882530.1	KF882521.1
KF882593.1	MG272177.1	KF882550.1	KF882556.1	KF882522.1
KF882588.1	MG272178.1	KF882550.1	KF882557.1	KF882520.1
KF882589.1	MG272179.1	KF882519.1	KF882543.1	KX362382.1
KF882594.1	MG272180.1	KF882535.1	KF882547.1	KX362393.1
KF882595.1	MG272181.1	KF882566.1	KF882545.1	KX362404.1
KF882523.1	MG272163.1	KF882542.1	KF882563.1	KX362415.1
KF882517.1	MG272164.1	KF882528.1	KF882549.1	
KF882539.1	MG272165.1	KF882558.1	KF882544.1	
KF882576.1	MG272166.1	KF882518.1	KF882525.1	
KF882538.1	MG272167.1	KF882526.1	KF882532.1	

Table S11. Genbank accession numbers of swine VP7 used in phylogenetic analysis with Buryat15.

KR052719.1	JQ043772.1	JQ043779.1	MF522282	MF522334	MF522386
KX376976.1	JQ043750.1	JQ043781.1	MF522283	MF522335	MF522387
KJ613649.1	MG272192.1	JQ043783.1	MF522284	MF522336	MF522388
KJ613650.1	MG272193.1	JQ043803.1	MF522285	MF522337	MF522389
KJ613646.1	MG272194.1	JQ043804.1	MF522286	MF522338	MF522390
AB490441.1	MG272195.1	JQ043761.1	MF522287	MF522339	MF522391
AB490442.1	MG272196.1	JQ043762.1	MF522288	MF522340	MF522392
AB490444.1	MG272197.1	JQ043775.1	MF522289	MF522341	MF522393
AB490445.1	MG272198.1	JQ043776.1	MF522290	MF522342	MF522394
AB490443.1	MG272199.1	JQ043788.1	MF522291	MF522343	MF522395
AB490446.1	MG272200.1	JQ043808.1	MF522292	MF522344	MF522396
AB490447.1	MG272201.1	JQ043773.1	MF522293	MF522345	MF522397
AB490448.1	MG272202.1	JQ043807.1	MF522294	MF522346	MF522398
AB490449.1	MG272203.1	JQ043765.1	MF522295	MF522347	MF522399
AB490439.1	MG272185.1	JQ043765.1	MF522296	MF522348	MF522400
AB490440.1	MG272186.1	JQ043780.1	MF522297	MF522349	MF522401
AB490450.1	MG272187.1	JQ043785.1	MF522298	MF522350	MF522402
AB490451.1	MG272188.1	JQ043786.1	MF522299	MF522351	MF522403
AB490452.1	MG272189.1	JQ043787.1	MF522300	MF522352	MF522404
AB490453.1	MG272190.1	JQ043797.1	MF522301	MF522353	MF522405
AB490454.1	MG272191.1	JQ043799.1	MF522302	MF522354	MF522406
AB490417.1	JQ043791.1	JQ043800.1	MF522303	MF522355	MF522407
AB490417.1	JQ043795.1	JQ043798.1	MF522304	MF522356	MF522408
AB490419.1	JQ043796.1	JQ043800.1	MF522305	MF522357	MF522409
AB490422.1	MG272204.1	JQ043798.1	MF522306	MF522358	MF522410
AB490423.1	MG272206.1	JQ043757.1	MF522307	MF522359	MF522411
AB490424.1	JQ043767.1	JQ043792.1	MF522308	MF522360	MF522412
AB490425.1	JQ043771.1	JQ043793.1	MF522309	MF522361	MF522413
AB490426.1	JQ043774.1	JQ043794.1	MF522310	MF522362	MF522414
AB490427.1	JQ043777.1	KX362394.1	MF522311	MF522363	MF522415
AB490420.1	JQ043782.1	KX362405.1	MF522312	MF522364	MF522416
AB490428.1	JQ043784.1	KX362416.1	MF522313	MF522365	MF522417
AB490429.1	JQ043789.1	KX362427.1	MF522314	MF522366	MF522418
AB490430.1	JQ043790.1	MF522263	MF522315	MF522367	MF522419
AB490432.1	JQ043801.1	MF522264	MF522316	MF522368	MF522420
AB490433.1	JQ043802.1	MF522265	MF522317	MF522369	MF522421

(Table S11 continued)

AB490431.1	JQ043805.1	MF522266	MF522318	MF522370	MF522422
AB490434.1	JQ043805.1	MF522267	MF522319	MF522371	MF522423
AB490436.1	JQ043753.1	MF522268	MF522320	MF522372	MF522424
AB490435.1	JQ043809.1	MF522269	MF522321	MF522373	MF522425
AB490421.1	JQ043810.1	MF522270	MF522322	MF522374	MF522426
AB490437.1	JQ043811.1	MF522271	MF522323	MF522375	MF522427
AB490438.1	JQ043812.1	MF522272	MF522324	MF522376	MF522428
AB490418.1	JQ043813.1	MF522273	MF522325	MF522377	MF522429
JQ043756.1	JQ043815.1	MF522274	MF522326	MF522378	MF522430
JQ043758.1	JQ043754.1	MF522275	MF522327	MF522379	MF522431
JQ043759.1	JQ043755.1	MF522276	MF522328	MF522380	MF522432
JQ043814.1	JQ043760.1	MF522277	MF522329	MF522381	MF522433
JQ043748.1	JQ043764.1	MF522278	MF522330	MF522382	MF522434
JQ043763.1	JQ043768.1	MF522279	MF522331	MF522383	MF522435
JQ043749.1	JQ043769.1	MF522280	MF522332	MF522384	MF522436
JQ043770.1	JQ043778.1	MF522281	MF522333	MF522385	
